# Emergency Department Management of COVID-19: An Evidence-Based Approach

**DOI:** 10.5811/westjem.2020.8.48288

**Published:** 2020-09-25

**Authors:** Nicholas M. McManus, Ryan Offman, Jason D. Oetman

**Affiliations:** Mercy Health – Muskegon, Department of Emergency Medicine. Muskegon, Michigan; Michigan State University College of Osteopathic Medicine, Department of Osteopathic Medical Specialties, East Lansing, Michigan

## Abstract

The novel coronavirus, SARs-CoV-2, causes a clinical disease known as COVID-19. Since being declared a global pandemic, a significant amount of literature has been produced and guidelines are rapidly changing as more light is shed on this subject. Decisions regarding disposition must be made with attention to comorbidities. Multiple comorbidities portend a worse prognosis. Many clinical decision tools have been postulated; however, as of now, none have been validated. Laboratory testing available to the emergency physician is nonspecific but does show promise in helping prognosticate and risk stratify. Radiographic testing can also aid in the process. Escalating oxygen therapy seems to be a safe and effective therapy; delaying intubation for only the most severe cases in which respiratory muscle fatigue or mental status demands this. Despite thrombotic concerns in COVID-19, the benefit of anticoagulation in the emergency department (ED) seems to be minimal. Data regarding adjunctive therapies such as steroids and nonsteroidal anti-inflammatories are variable with no concrete recommendations, although steroids may decrease mortality in those patients developing acute respiratory distress syndrome. With current guidelines in mind, we propose a succinct flow sheet for both the escalation of oxygen therapy as well as ED management and disposition of these patients.

## INTRODUCTION

It took just over two months for the novel coronavirus, SARs-CoV-2 to be declared a global pandemic by the World Health Organization (WHO). In the immediate week following this announcement, more than 400 papers were published pertaining to COVID-19. Just two months later, this number had increased to over 2000 releases per week in the literature.^1^ Keeping up with ever-changing information can be quite difficult. The purpose of this clinical review is to provide the emergency physician (EP) with a summary of current literature and supporting societal guidelines relevant to the management of the COVID-19 patient in the emergency department (ED). Finally, we propose an ED-based algorithm for the work-up and initial management of patients with suspected COVID-19 infections.

## METHODS

We systematically searched the PubMed, LitCovid, Ovid, Cochrane Library, MEDLINE, Google Scholar, and Embase for literature related to “COVID-19,” “SARS-CoV-1,” and “SARS-CoV-2.” We included retrospective studies, case reports, case series, systematic reviews, meta-analyses, and clinical guidelines from the Society of Critical Care Medicine (SCCM), the Surviving Sepsis Campaign (SSC), the National Institutes of Health (NIH), and the European Society of Intensive Care Medicine (ESICM). We included relevant literature if it contained data on epidemiological characteristics, biomarkers, imaging, oxygenation and ventilation management, procedural aerosolization, pathology reports, hematologic abnormalities, and treatment outcomes related to care commonly seen in the ED).

## DISCUSSION

### Risk Stratification

Risk stratification in the ED can be difficult for a novel virus such as SARS-CoV-2 as we do not have the luxury of years of research and understanding that we are offered with most disease processes. Decompensation of the otherwise well appearing COVID-19 patient can occur rather rapidly as many patients develop early lung injury and hypoxia before clinical deterioration is appreciated.^2^ The ability of the EP to identify features that recognize those patients most at risk for clinical deterioration would be ideal. While many risk-stratification models have been proposed in response to COVID-19, most lack COVID-19-specific data, mainly focus on in-hospital mortality. and lack validation in the literature.^3^–^6^

### National Institutes of Health (NIH) Definition of Disease Severity

Currently, evidence-based practices support using epidemiological, laboratory, radiographic, and clinical features to help us determine who is at risk for decompensation.^7^ The NIH describes a mild clinical course as those with various symptoms (eg, fever, fatigue, cough, myalgias, headache) but without dyspnea and with normal imaging.^7^ There is insufficient data for the NIH panel to recommend specific lab evaluation or treatment modalities in patients fitting this profile.^7^ Based on current evidence, considerations should include discharge home with recommendations of antipyretics, hydration, and rest with self-isolation until afebrile for 72 hours without the need for antipyretics and improving symptoms.^7^ Patients with moderate disease are defined as those with evidence of lower respiratory tract pathology based on imaging or clinical assessment, but still have pulse oximetry readings greater than 93%.^7^ These patients should be admitted for close observation. Empiric antibiotics for community-acquired pneumonia should be considered if a bacterial pneumonia or sepsis is suspected.^7^ The NIH classification of ***severe*** disease includes those with a respiratory rate greater than 30; blood oxygen saturation level equal to or less than 93% on room air, a ratio of arterial oxygen partial pressure to fractional inspired oxygen < 300 or > 50% of lung involvement on imaging.^7^ These patients will require supportive oxygen therapy and hospital admission.^7^

### Epidemiological Risk Factors as Predictors of Disease Severity

The largest case series assessing epidemiological risk factors includes a 72,314-patient report from the Chinese Center for Disease Control and Prevention.^8^ They noted independent risk of death in patients was 10.5% for cardiovascular disease, 7.3% for diabetes mellitus, 6.3% for chronic respiratory diseases, 6% for hypertension, and 5.6% for underlying malignancy. This is compared to an overall case fatality rate of 0.9% in those without these comorbid conditions. A meta-analysis of six studies assessing a total of 1558 patients showed that hypertension, diabetes, chronic obstructive pulmonary disease (COPD), cardiovascular disease, and cerebrovascular disease were all independent risk factors associated with increased disease severity and intensive care unit (ICU) admission.^9^ They found no association between COVID-19 risk and liver disease, renal disease, or malignancy. In a case series of 700 patients hospitalized with COVID-19 in New York City, the most common comorbidities among patients requiring hospitalization include hypertension (56.6%), obesity (41.7%), and diabetes (33.8%) with 88% of patients having more than one comorbidity.^10^

An article published by Guo and colleagues showed that COVID-19 patients with diabetes but without other comorbidities were at an independently high risk of severe pneumonia, uncontrolled inflammatory response, and hypercoagulable state.^11^ Serum D-dimer, interleukin (IL)-6, C-reactive protein (CRP), and ferritin were significantly higher in patients with diabetes mellitus showing susceptibility to rapid deterioration in COVID-19. A retrospective observational study of 1122 adults with laboratory-confirmed COVID-19 showed a mortality rate of 41.7% in diabetic patients with uncontrolled hyperglycemia defined as greater than two blood glucose readings greater than 180 milligrams per deciliter within a 24-hour period.^12^

Several studies have linked obesity and a body mass index (BMI) greater than 30 kilograms (kg) per meter squared (m^2^) with increased risk of mechanical ventilation, severe pneumonia, and death associated with COVID-19.^13^,^14^ Further, a BMI greater than 30 kg/m^2^ in those younger than 60 has been noted to be an independent risk factor with a twofold higher rate of acute care and ICU admission when compared to those with a BMI less than 30 kg/m^2^.^15^

In the March 2020 Morbidity and Mortality Report from the US Centers for Disease Control and Prevention (CDC), patients above the age of 65 had a particularly significant increased risk of death when compared to their younger counterparts with up to 80% of deaths occurring in those over the age of 65.^16^

Finally, a single-center study of 1193 patients in Lombardia, Italy, showed patients on biologics had a higher rate of hospitalization, but this was not associated with an increased risk of ICU admission or death.^17^ Until more is known, most sources including the CDC recommend close monitoring of immunocompromised patients, those with untreated or uncontrolled human immunodeficiency virus, and those on biologics. This recommendation is based on mostly anecdotal concern that these patients may remain infectious for longer periods of time.

The EP should maintain a baseline level of caution when determining disposition of these patients, especially in patients with more than one comorbid condition. In a prediction model from Wang et al, hypertension, advanced age, and coronary heart disease, their model appears to confer the highest risk of in-hospital mortality with an area under the curve of 0.88; sensitivity, 92.31%; specificity, 77.44%; and negative predictive value (NPV) of 99.34%).^18^

### Lab Values as Predictors of Disease Severity

Many serum biomarkers have been studied with COVID-19 infections. Alanine transaminase (ALT) and aspartate aminotransferase (AST) tend to be elevated and albumin low. Elevations in lactate dehydrogenase (LDH), CRP, procalcitonin, and abnormalities in coagulation parameters such as ferritin, D-dimer, fibrinogen, activated partial thromboplastin time and prothrombin time all tend to be elevated in patients with poor progression of disease.^19^ Measurements of these values should be considered in any patient with moderate to severe disease for their prognostic value. It is important to note that while guidelines recommend consideration in obtaining these markers, they are not considered part of standard care.^7^ While many of these lab values are non-specific to COVID-19, they may serve as a tool for the EP until more robust prediction models are further studied and validated in the future.

#### Absolute Lymphocyte Count (ALC)

An ALC less than 0.8×10^9^ per liter (L) has been consistently shown to correlate with disease severity, ICU admission, and death.^19^ Those with values greater than 1 ×10^9^/L tend to have a milder disease process, and values below this could perhaps help identify those at risk for disease progression. A summary of literature addressing ALC has been summarized in Appendix 1.

#### Neutrophil to Lymphocyte Ratio (NLR)

An elevated neutrophil count has been shown to correlate with disease severity. However, an absolute value to determine severity is not as apparent in current literature. The NLR may offer greater clinical insight. Normal values of the NLR range between 0.78–3.53 with a mean value of 1.65.^20^ A study by Xia et al of 10 patients identified that those with non-severe cases had a calculated NLR in the range of 1.29–6.14, while all three patients with more severe cases had values greater than 10.^21^ An elevated NLR has been show to predict poor outcomes in COVID-19 with a specificity of 63.6% and a sensitivity of 88%.^22^ For each increase in NLR tertile, hospital mortality increases by 8%.^23^ A summary of literature addressing neutrophil count has been summarized in Appendix 1.

#### D-dimer

An elevated D-dimer has been shown to be an independent marker of unfavorable disease progression in multiple studies.^24^–^31^ In the retrospective study from Zhou et al 81% of patients who died had a D-dimer greater than 1 microgram per milliliter (μg/mL) on admission. In a retrospective study of 343 hospitalized patients in Wuhan, China, the optimum cutoff value for D-dimer to predict all-cause death was 2.0 μg/mL using receiver operating characteristic curve with a sensitivity and specificity of 92.3% and 83.3%, respectively.^32^ In fact, a prospective study of 183 consecutive patients by Tang and colleagues showed that 71.4% of non-survivors demonstrated disseminated intravascular coagulation (DIC) during their hospital stay, while only 0.6% of survivors did. While an optimum cutoff has not been validated, a twofold increase in values has consistently been shown to predict disease severity in numerous studies.^25^,^28^,^29^,^33^–^38^ An elevated D-dimer used for risk stratification does not currently warrant routine investigation for acute venous thromboembolism (VTE) in absence of clinical manifestations or other supporting information in favor of VTE.^39^ A summary of the literature addressing D-dimer has been summarized in Appendix 2.

#### Lactate Dehydrogenase (LDH**)**

In the previously discussed study by Zhou and colleagues, an LDH greater than 245 was seen in 98% of all patients who did not survive, with an odds ratio for in-hospital mortality of 45.43.^24^ However, this elevation was also seen in 54% of those who survived. While an elevated LDH has shown an increased association with those requiring ICU admission and predicting in-hospital mortality in multiple studies, a normal value has also been shown to predict those who ultimately had a more mild to moderate disease process.^24^,^25^,^29^,^30^,^36^,^37^,^40^,^41^ A summary of the literature addressing LDH has been summarized in Appendix 3.

#### C-reactive Protein (CRP)

CRP is non-specific and frequently elevated in patients with mild disease.^28^,^36^–^38^,^42^,^43^ However, the degree of increase has been associated with worse outcomes and in-hospital mortality as levels increase greater than 100 milligrams (mg)/L. Less significant elevations (50–75 mg/L) were seen in patients ultimately discharged home.^44^ A summary of the literature addressing CRP has been summarized in Appendix 3.

#### Ferritin

Ferritin is another nonspecific marker with elevations seen in up to 63–80% of COVID-19 patients admitted to the hospital.^24^,^45^ Ferritin levels greater than 300 nanograms (ng)/mL have been associated with in-hospital mortality at an odds ratio of 9.10. A recent retrospective, multicenter study of 150 COVID-19 cases in Wuhan showed a mean elevation of 1297.6 ng/mL in non-survivors versus 614.0 in survivors.^44^ A summary of the literature addressing ferritin has been summarized in Appendix 3.

#### Creatine Kinase (CK)

Creatine kinase (CK) appears to be elevated in a minority of COVID-19 patients regardless of severity.^25^,^38^,^45^,^46^ In the Zhou et al study, a CK greater than 185 units (U)/L was seen in 21% of non-survivors and 9% of survivors with an in-hospital mortality odds ratio of 2.56.^24^ Certain patients may benefit from having CK levels checked, especially those with significant myalgias, as COVID-19-related myositis has been described in the literature.^47^

### Imaging as a Marker of Disease Severity

There is a lack of evidence in published literature to suggest that laterality of infiltrates on imaging accurately correlates with disease severity. In a retrospective cohort study out of Wuhan, bilateral infiltrates were seen in 72% of survivors and 83% of non-survivors.^24^ However, multiple studies have shown bilateral involvement in as high as 91–100% of all patients admitted to various hospitals across China, regardless of disease severity.^25^,^29^,^37^,^41^,^42^

In a multinational consensus statement from the Fleischner Society, chest imaging is recommended in those patients with mild symptoms and any risk factors of disease progression, in all patients with moderate to severe features, or when rapid COVID-19 testing is not available.^48^ Current guidelines from the American College of Radiology (ACR) recommends considering portable chest radiographs (CXR) to avoid bringing patients into radiography rooms and recommends against computed tomography (CT) unless clinically indicated for another reason.^49^

Bedside lung ultrasound (LUS) may offer some advantages in the ED for patients with suspected COVID-19.^50^ A recently published article of 391 patients showed that LUS had a higher sensitivity when compared to CXR in patients diagnosed with COVID-19 pneumonia.^51^ Considering COVID-19 reverse transcription polymerase chain reaction (RT-PCR) has a sensitivity as low as 60–70% and CT findings can be delayed, LUS findings may add increased sensitivity to diagnosis.^52^ Further, ultrasound has safety advantages including absence of radiation, low cost, and rapid bedside availability.^53^

Focal B-lines in the posterior and inferior lung fields appear to be the primary finding.^54^ As disease progresses, the pleura becomes thickened and irregular with multifocal or confluent B-lines.^54^,^55^ In a study of 20 patients with moderate to critical severity COVID-19 pneumonia, pleural line abnormalities and B-lines were present in 100% of study participants.^56^ LUS findings have been shown to highly correlate with findings on CT.^54^

### Management of The Critically Ill Adult

Current guidelines for the management of the critically ill adult with COVID-19 have been issued by the SCCM, the SSC, the NIH, and the ESICM. These guidelines are quite similar, if not identical, in regard to most recommendations and will be summarized here.^7^,^57^,^58^

#### Hemodynamic Support

Current guidelines favor a conservative approach to fluids in these patients. Utilization of early vasopressors is recommended to keep a mean arterial pressure (MAP) of 60–65 millimeters of mercury (mm Hg), although this is based on low quality of available evidence.^7^,^57^,^58^ Instead, it is based on the historical approach to patients with ARDS while in the ICU, largely after initial resuscitation in the ED, suggesting that a conservative approach to fluids leads to more ventilator-free days and shorter ICU stays, but has failed to show mortality benefit.^59^–^63^ The initial resuscitation fluid should be a buffered/balanced crystalloid, avoiding colloidal fluid and albumin.^7^,^57^,^58^ Guidelines are consistent in their recommendation of norepinephrine as the first-line agent and suggests adding vasopressin as a second-line agent early instead of titrating norepinephrine to higher doses.^7^,^57^,^58^ Epinephrine or vasopressin is the recommended first-line agent if norepinephrine is not available.

Dobutamine should be considered a second-line agent after norepinephrine only if there is evidence of cardiac dysfunction and persistent hypoperfusion.^7^,^57^,^58^ Dopamine should be avoided if norepinephrine is available due to an increased risk of arrhythmias.^7^,^57^,^58^,^62^ In patients with refractory shock despite vasopressors, administration of stress-dose steroids (ie, intravenous hydrocortisone 200 mg per day) are recommended; however, this has not specifically been studied in COVID-19.^7^,^57^,^58^,^63^,^64^

#### Oxygen and Ventilation

Early discussion of hypoxic patients with COVID-19 prioritized intubation based on the hypothetical risk of patient self-induced lung injury resulting from excessive intrathoracic negative pressure from strong respirator effort and aggressive positive pressure ventilation strategies.^65^–^70^ Further, data suggest that ARDS patients with severe hypoxemic respiratory failure who received noninvasive ventilation (NIV) had a higher ICU mortality.^71^ Limited data from the severe acute respiratory syndrome and Middle East respiratory syndrome outbreaks show a high failure rate of NIV coupled with concern of virus aerosolization made early intubation for all who were hypoxic seem more veracious.^65^–^67^ Currently there is a lack of evidence identifying the ideal time of intubation, and this area would benefit from additional research.

The FLORALI trial randomly assigned patients who had acute hypoxemic respiratory failure to either high-flow oxygen therapy or standard oxygen therapy delivered through a face mask, or noninvasive positive-pressure ventilation.^72^ There was no significant difference in the intubation rates between groups; however, there was a significant difference in favor of high-flow oxygen in 90-day mortality. An unblinded, retrospective study of hospitalized COVID-19 patients concluded that high flow nasal oxygen (HFNO) therapy provided more patient comfort and was non-inferior to NIV for intubation rate.^73^ The ANZICS guidelines on COVID-19 state that HFNO appears to be at least non-inferior to NIV and may even offer survival benefit.^74^ HFNO is a recommended therapy for hypoxia associated with COVID-19 disease, as long as staff are wearing optimal airborne personal protective equipment where the risk of airborne transmission to staff is low.^57^,^68^

Early case reports described COVID-19 patients presenting with ARDS and a ventilatory management strategy typically employed in ARDS was recommended by the WHO and SCCM.^57^,^68^ However, observations from Italy described a subset of patients who met Berlin criteria for ARDS and presented with rather profound hypoxemia without the expected degree of observed dyspnea.^75^,^76^ This observation suggests that there may be more than one phenotypic presentation of COVID-19-induced lung injury.

Those with “type-H” phenotype present with a clinical picture characteristic of typical ARDS (low compliance, high lung weight and high positive end-expiratory pressure [PEEP] response).^67^,^75^ In patients with COVID-19 and ARDS, using lower tidal volumes (4–8 mL/kg predicted body weight), lower inspiratory pressures (plateau pressure < 30 centimeters of water (cmH20) and higher PEEP for recruitment is currently recommended by the SCCM and WHO.^57^,^68^

Those with the observed “type-L” phenotype frequently have minimal dyspnea and remain alert and conversational despite the degree of observed hypoxia.^77^ This process is thought to be due to a loss of hypoxic vasoconstriction and impaired regulation of pulmonary blood flow leading to a ventilation-perfusion (V/Q) mismatch.^76^ In these patients, lung compliance remains relatively normal and can accept larger tidal volumes (7–8 mL/kg ideal body weight) to help avoid reabsorption atelectasis and hypercapnia from hypoventilation.^67^,^76^,^77^ Recruitability is minimal and, therefore, a high PEEP strategy is unlikely to improve oxygenation and may be detrimental.^66^,^67^,^76^ HFNO and prone positioning may help redistribute pulmonary perfusion and improve the V/Q mismatch.^76^ In patients who are alert, allowing them to self-prone has been shown to improve oxygenation and is a reasonable approach for those not otherwise requiring intubation.^67^

**Figure 1:**
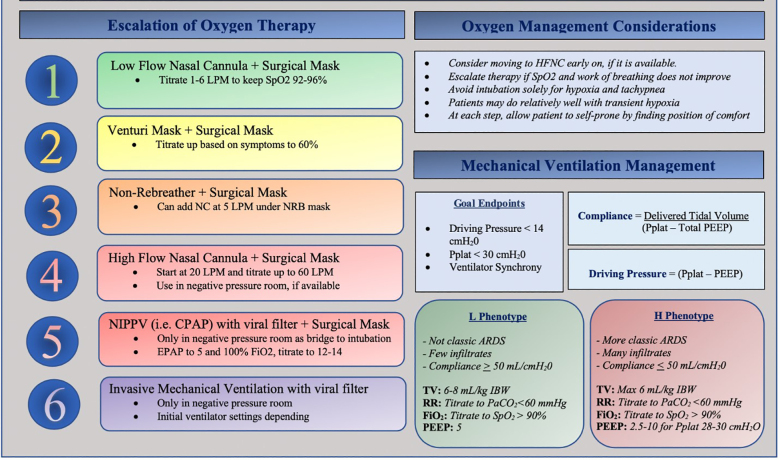
Respiratory management in coronavirus 2019 disease. *LPM*, liters per minute; NRB, non-rebreather mask; *NIPPV*, noninvasive positive pressure ventilation; *CPAP*, continuous positive airway pressure; *EPAP*, expiratory positive airway pressure; *SpO_2_*, peripheral capillary oxygen saturation; *RR*, respiratory rate; *Pplat*, plateau pressure; *PEEP*, positive end-expiratory pressure.

This phenotype model is untested and there is a paucity of societal guidelines for patients with preserved compliance requiring mechanical ventilation. We believe a blanket ARDS ventilatory strategy for all patients could have detrimental consequences.^75^ Given the variable differences in observed lung compliance in clinical presentations of COVID-19, it is reasonable to consider a targeted ventilatory strategy unique to the observed lung mechanics and not simply the degree of hypoxia (Figure 1).

#### Surface Stability and Aerosolization of SARS-CoV-2

While the presence of viral particles does not confer transmission, it certainly supports our need to exercise caution to maximize protection to ourselves and our staff. A meta-analysis of 10 studies published in the *Journal of Infectious Disease* reported that shows droplets from coughs and sneezes can travel up to eight meters, with SARS-CoV-2 detected in the air up to 3–5 hours after aerosolization.^78^,^79^ In a study from the University of Nebraska Medical Center, SARS-CoV-2 RNA has been isolated throughout patient rooms, their personal items, in the air ducts, and even outside in the hallway suggesting aerosolized transmission.^80^

Exhaled air dispersion during high-flow nasal cannula therapy was compared to continuous positive airway pressure (CPAP) in a study by Hui et al.^81^ The mean air dispersion was up to 172 +/− 33 mm along the sagittal plane via HFNO at 60 L/minute (min), and similar leakage distances could be detected up to 264 and 332 mm for CPAP used up to 20 cm H20. A properly fitted, heated HFNO appears to be the safer option in regard to dispersion of aerosols, and therefore may be the safer option to minimize risk to staff. The Vapotherm study performed a simulation with HFNO and a surgical mask on the patient to assess dispersion.^82^ The results showed that by placing a simple mask over a patient receiving high-flow therapy, 87.2% of particles were effectively filtered. Those particles that did leak around the mask,had a final path length of less than one meter.

#### Aerosolization Risk Based on Oxygen Modality

**Figure 2:**
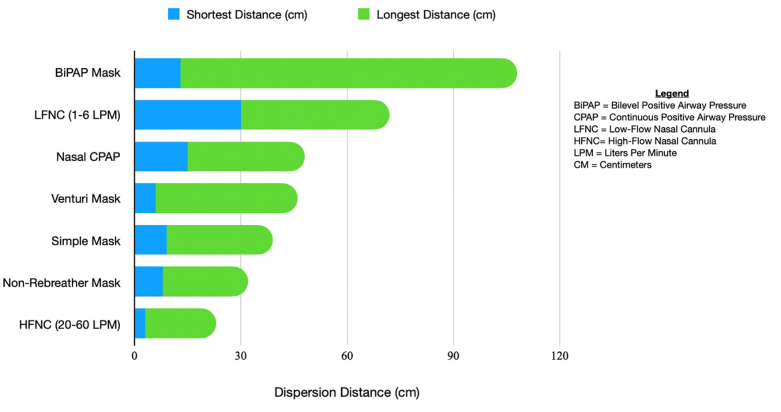
Oxygen modality dispersion distances. (Li et al; Whittle et al; Hui et al)

HFNO at a maximal flow rate of 60 L/minPM actually has a lower dispersion distance than a non-rebreather or venturi mask.^83^ A study by Whittle et al showed NIV had the longest range of dispersal at 85–95 cm. Nebulized medications were similar at 80 cm.^84^ HFNO has an average of approximately 5–17 cm with low flow nasal cannula reaching up to 40 cm in some studies. A summary of dispersion distances in relevant literature is shown in Figure 2.

### Thrombotic and Thromboembolic Disease

Patients with COVID-19 are at an increased risk of VTE. Current documented rates of incidental VTE in hospitalized patients with COVID-19 ranges from 20–69%, despite the use of pharmacological thromboprophylaxis.^85^–^90^ The DIC observed in severe COVID appears to be solely prothrombotic, and patients with the most severe disease at most risk.^88^ Nearly 15% of thrombotic events are asymptomatic.^91^

#### Diagnosing Incidental Venous Thromboembolism

In a study of 81 patients with COVID-19 infections, a D-dimer greater than 1.5 μg/mL had a sensitivity of 85.0%, a specificity of 88.5% with a NPV of 94.7% at predicting VTE.^86^ However, this is a rather small study and lacks validation. While a threshold value for an elevated D-dimer in COVID-19 has not yet been established, a significant elevation has shown to correlate with the presence of VTE and an increase in mortality.^92^ The *Journal of the American College of Cardiology* (*JACC*) panel recommends against routine screening for VTE and recommends against pursuing an elevated D-dimer when it is being used for risk stratification.^39^

#### Patients with Mild COVID-19 Treated as Outpatient

The *JACC* panel does not recommend routine use of prophylactic anticoagulation as its role has not yet been well established in the literature.^39^ Patients who are on chronic antiplatelet agents or anticoagulants should be encouraged to continue taking these medications. For patients on Vitamin K antagonists who will be unable to get routine international normalized ratio measurements, switching them to a direct oral anticoagulant or low molecular weight heparin is a reasonable option.

#### Patients with Moderate to Severe COVID-19 Requiring Hospitalization

Patients with ARDS secondary to COVID-19 are at a higher risk of thrombosis when compared to non-COVID-19 ARDS patients.^85^ Further, the development of incidental VTE in patients with severe COVID-19 is lower in those treated with therapeutic dose anticoagulation over prophylactic dosing.^90^ Based on a paucity of evidence at the time of publication, the majority of *JACC* panel members recommend prophylactic anticoagulation for hospitalized COVID-19 patients without a diagnosis of VTE, while a minority of the panel gives consideration to intermediate- or full-dose anticoagulation.^39^ Some hospital systems are currently using a higher prophylactic dose such as enoxaparin 1 mg/kg once daily or enoxaparin 0.5 mg/kg twice daily.^92^ We anticipate future guideline adjustments in regard to therapeutic anticoagulation in select patients as more robust evidence on its impact on mortality emerges.

### Adjunctive Therapy

#### Antipyretics and NSAIDs

Controversy surrounds the use of nonsteroidal anti-inflammatory drugs (NSAID) in COVID-19 stemming from a correspondence published on March 11, 2020, in the *Lancet* describing a theoretical risk of worsening infection through increased ACE-2 expression with ibuprofen based on animal studies.^93^ Initial WHO recommendations were to avoid ibuprofen based on this concern, and on March 19 the US Food and Drug Administration issued a statement suggesting a lack of scientific evidence in connection with NSAIDs and worsening COVID-19 symptoms.

When the SSC released its guidelines on March 27, they acknowledged the debate on NSAIDs use for fever, and recommended the use of acetaminophen/paracetamol over NSAIDs until more data becomes available. On April 19, the WHO released a systematic review of 73 studies of adults and children with viral respiratory infections, including COVID-19, MERS, and SARS and concluded that, “At present there is no evidence of severe adverse events, acute health care utilization, long-term survival, or quality of life in patients with COVID-19, as a result of the use of NSAIDs.”^94^ The NIH guidelines were initially released on April 21 and recommended there be no difference in the use of antipyretics (acetaminophen or NSAIDs) in patients with COVID-19.^7^ It is important to point out that it has been well documented outside of COVID-19 that fever control has not been shown to reduce the risk of death or ICU length of stay in a critically ill adult.^57^

#### Steroids

Initial concerns in regard to the use of corticosteroids in COVID-19 were based on studies specific to SARS-CoV-1 showing prolonged viral shedding with early corticosteroid treatment and an increased risk of adverse effects such as steroid-induced psychosis, avascular necrosis osteoporosis, and diabetes without an apparent mortality benefit.^95^–^98^ It is important to note that these early studies focused on rather high doses of steroids and despite prolonged viral shedding (12 days vs eight days), those who received corticosteroids were less likely to clinically deteriorate.^95^,^99^ A 2020 study using low-dose corticosteroids (mean dose approximately 40 mg methylprednisolone daily) in patients with COVID-19 showed steroids had no impact on viral shedding.^100^

A 2016 retrospective review of 5327 patients from the SARS-CoV-1 database in China showed that patients initially treated with an average of 80 mg methylprednisolone daily had a lower mortality with a hazard ratio (HR) of 0.47.^101^

Results from randomized trials in regard to steroids in ARDS from non-coronavirus causes have shown mixed outcomes. High dose (30 mg/kg every 5–6 hours for 24 hours) failed to show improvement in mortality or pulmonary function and was associated with an increased rate of secondary infection.^102^,^103^ However, a study looking at a more prolonged and lower dose course (2 mg/kg/day for two weeks, and then tapered for a total of 32 days of treatment) showed improvement in lung injury and a reduced hospital-associated mortality when compared to placebo (12% vs 62%, respectively) in patients with severe ARDS who failed to improve by seven days.^104^

Data specific to COVID-19 and ARDS is limited. A retrospective study of 201 COVID-19 patients in Wuhan showed that of the patients who developed ARDS, those who received methylprednisolone in some fashion had a decreased risk of death with HR of 0.38.^30^ Another retrospective study of 46 patients out of Wuhan showed that early, low-dose and short-term corticosteroid use (1–2 mg/kg/d for 5–7 days), was associated with faster wean off supplemental oxygen (8.2 days vs 13.5 days) and faster improvement of infiltrates on CXR.^105^ However, neither study was a randomized controlled trial (RCT), and improvements seen could have been from variations in other aspects of treatment strategies. A recent meta-analysis from Ye et al of seven RCTs of non-COVID-19-related ARDS and one small cohort study of COVID-19-related ARDS showed that corticosteroids may reduce mortality with a risk ratio of 0.72.^106^ In the meta-analysis from Ye et al, data from two observational studies showed that corticosteroid use in patients with COVID-19 infection but without ARDS resulted in an increase in mortality with a HR of 2.30 and a mean difference of 11.9% more.^106^

In summary, corticosteroids may decrease mortality in COVID-19 patients with ARDS. The SSC recommends steroids for mechanically ventilated patients with COVID-19 and evidence of ARDS or for refractory shock despite vasopressors.^57^ The NIH guidelines recommend a case-by-case approach to steroids in critically ill patients with ARDS, citing insufficient evidence to recommend blanket use for all mechanically vented patients with ARDS.^7^ The most recent update of the Infectious Disease Society of America guidelines recommend dexamethasone 6 mg daily for up to 10 days in hospitalized patients with pulse oximetry readings ≤ 94% on room air. If dexamethasone is unavailable, methylprednisolone 32 mg, or prednisone 40 mg may be used.^7^,^107^ Guidelines do not currently recommend the use of steroids in patients with COVID-19 in the absence of hypoxia or ARDS unless they have a history of chronic underlying lung disease (ie, asthma, COPD, or pulmonary fibrosis).

For patients on chronic oral or inhaled corticosteroids, these should not be discontinued, and stress-dose steroids may be indicated on a case-by-case basis.^7^ Specific to pregnancy, betamethasone and dexamethasone are known to cross the placenta and should therefore be reserved for situations when fetal benefit is needed. However, other systemic corticosteroids do not cross the placenta, and pregnancy status alone should not be a reason to restrict their use.^7^

#### Antimicrobials

A recent meta-analysis of patients admitted with COVID-19 reported 72% receive empiric antimicrobials, while only 8% of patients develop a bacterial or fungal co-infection.^108^ The SCCM guidelines recommend empiric antibiotics for mechanically ventilated patients with COVID-19 and respiratory failure based on low-quality evidence.^57^ The NIH has stated there is insufficient data to recommend empiric broad-spectrum antibiotics in the absence of another indication.^7^ If empiric antibiotics are initiated, they should be de-escalated as soon as clinically possible.

Numerous studies done in vitro have reported antiviral and anti-inflammatory effects of azithromycin, although the exact mechanism of antiviral activity is unknown.^109^ There are currently no guideline recommendations in favor of azithromycin. Further, there is a theoretical possibility that doxycycline could have anti-inflammatory action against IL-6 and perhaps offer benefit in COVID-19.^110^ While these medications are frequently prescribed out of the ED, there are no specific societal guidelines recommending their use in COVID-19 at this time.

#### Inhaled Nitric Oxide

Inhaled nitric oxide (NO) is a pulmonary vasodilator with theoretic antiviral effects.^111^ In a 2004 study of 14 patients with SARS being treated in the ICU with noninvasive pressure support, NO use for three days was associated with improved oxygenation and a decrease in severity of infiltrates on imaging.^112^ As with most treatments, data with NO use in COVID-19 is lacking. Therefore, SSC and NIH guidelines recommend against routine pulmonary vasodilator use but recognize that a trial of inhaled NO as a rescue therapy is reasonable and should be discontinued if there is no rapid improvement in oxygenation.^7^,^57^

#### Renin-Angiotensin-Aldosterone System (RAAS) Inhibitors

Early reports suggested an association of severe COVID-19 with renin-angiotensin-aldosterone system (RAAS) antagonist use leading to advice to discontinue this medication.^113^ Three studies were recently published, with a total of 21,076 confirmed COVID-19 patients looking at RAAS inhibitors and risk of COVID-19. These studies did not demonstrate increased severity of illness with patients taking angiotensin-converting enzyme inhibitors, angiotensin II receptor blockers, calcium channel blocker, beta blocker, or thiazide diuretics.^114^,^115^ The Heart Failure Society of America, the American College of Cardiology and the American Heart Association released a joint statement recommending these medications be continued in patients who take them for chronic medical conditions unless for actions based on standard clinical practice.^116^

### Controversial Therapies

#### Aspirin

Currently, no guidelines specifically mention aspirin in their recommendations. Aspirin has a theoretical benefit for its antiplatelet, anti-inflammatory, and antipyretic effects. Studies have shown that aspirin has in vitro antiviral activity against influenza A, human rhinoviruses, and human cytomegalovirus.^117^,^118^ Further, indomethacin has been shown to have a potent antiviral activity against SARS-CoV-1.^119^ Multiple studies are currently enrolling and assessing the effects of aspirin in COVID-19 (NCT04365309, NCT04343001, NCT04363840, NCT04333407). Future research should focus on potential preventative effects of aspirin and its effects on disease severity, particularly in patients being discharged home from the ED.

## LIMITATIONS

This paper has a few notable limitations. First, with the large volume and rapid publication of literature on this previously unknown subject, most lack validation. Some articles regarding COVID-19 have been retracted after publication, although every effort has been made to be sure each citation was valid at the time of publication of this manuscript. Finally, only articles published in English were reviewed.

## CONCLUSION

**Figure 3:**
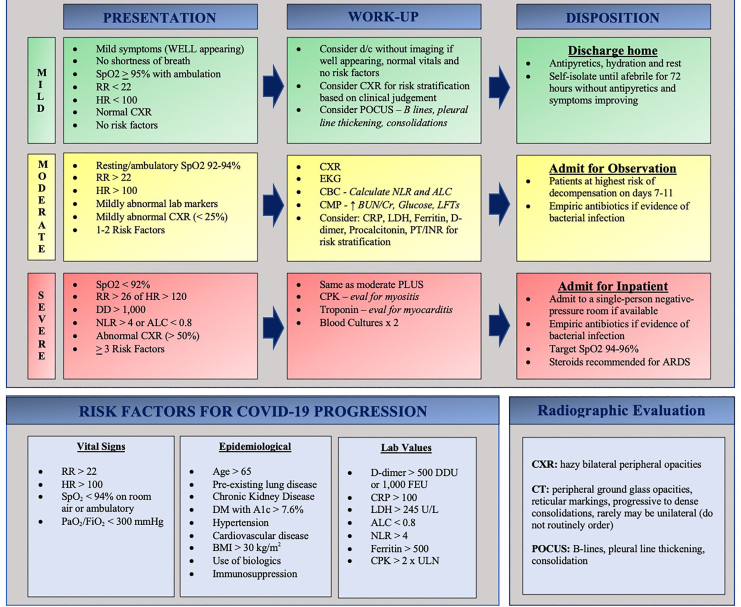
COVID-19 emergency department evaluation. *SpO_2_*, peripheral capillary oxygen saturation; RR, respiratory rate; *HR*, heart rate; *d/c*, discharge; *CXR*, chest radiograph; *US*, ultrasound; *POCUS*, point-of-care ultrasound; *EKG*, electrocardiogram; *CBC*, compete blood count; *NLR*, neutrophil to lymphocyte ratio; *ALC*, absolute lymphocyte count; *CMP*, comprehensive metabolic panel; *BUN*, blood urea nitrogen; *CR*, creatinine; *LFT*, liver function test; *CRP*, C-reactive protein; *LDH*, lactate dehydrogenase; *PT/INR*, prothrombin time/international normalized ratio; *CPK*, creatine phosphokinase; *ARDS*, acute respiratory distress syndrome; *PaO_2_ ;* partial pressure of oxygen; *FiO_2_ ;* fraction of inspired oxygen; *DM*, diabetes mellitus; *BMI*, body mass index; *CT*, computed tomography.

Evidence-based practice in the approach to COVID-19 is mercurial. Current literature focuses on the inpatient evaluation, treatment, and disposition of these patients. Interpretation and adaptation of current recommendations to patients in the ED is a crucial target for future literature. After our review of available literature, we have proposed an ED-specific flowsheet to assist clinicians during this time of medical ambiguity (Figure 3).

## Supplementary Information
