# Supplementary material for: Emergency Department Management of COVID-19: An Evidence-Based Approach
Source: West J Emerg Med. 2020 Sep 25;21(6):32–44. doi: 10.5811/westjem.2020.8.48288 (PMC7673887; doi:10.5811/westjem.2020.8.48288)
Supplement: Supplementary file 1 [file wjem-21-32-s001.docx]

| **Appendix 1a: Absolute Lymphocyte Count (ALC) x 10^9^/L** | | | | |
| --- | --- | --- | --- | --- |
| **Study** | **Outcomes Measured** | **Median value** | **95% CI** | **p-value** |
| **Zhou et al.^24^**  (N=191) | **Survivors** | *0.6* | *0.5–0.8* | *p<0.0001* |
|  | **Non-survivors** | *1.0* | *0.6–1.3* | *p<0.0001* |
| **Richardson, et al.^10^**  (N=5,700) | **Survivors** | *0.8-2.3* | *0.5-5.0* | *not documented* |
|  | **Non-Survivors** | *0.5* | *0.3-0.8* | *not documented* |
| **Huang, et al.^25^**  (N=41) | **No ICU care** | *1.0* | *0.7–1.1* | *p=0.0041* |
|  | **ICU care** | *0.4* | *0.2–0.8* | *p=0.0041* |
| **Wu, et al.^30^**  (N=201) | **Without ARDS** | *1.08* | *0.72-1.45* | *p<0.001* |
|  | **With ARDS (alive)** | *0.80* | *0.56-1.17* | *p=0.004* |
|  | **With ARDS (died)** | *0.59* | *0.48-0.74* | *p=0.004* |
| **Wang, et al.^29^**  (N=138) | **Non-ICU** | *0.9* | *0.6-1.2* | *p=0.03* |
|  | **ICU** | *0.8* | *0.5-0.9* | *p=0.03* |
| **Guan, et al.^40^**  (N=1,099) | **Non-severe** | *0.8* | *0.6-1.0* | *not documented* |
|  | **Severe** | *1.0* | *0.8-1.4* | *not documented* |
| **Wang, et al.^18^**  (N=296) | **Survivors** | *1.0* | *0.7-1.4* | *p=0.003* |
|  | **Non-survivors** | *0.7* | *0.5-1.0* | *p=0.003* |
| **Wang, et al.^18^**  (N=44) | **Survivors** | *0.9* | *0.7-1.2* | *p=0.048* |
|  | **Non-survivors** | *0.6* | *0.5-0.8* | *p=0.048* |
| **Yao, et al.^42^**  (N=108) | **Non-severe** | *1.41* | *1.01-1.77* | *p<0.001* |
|  | **Severe (alive)** | *0.79* | *0.64-0.95* | *p<0.001* |
|  | **Severe (died)** | *0.76* | *0.63-1.58* | *p<0.001* |
| **Wang, et al.^43^**  (N=65) | **Mild** | *0.9* | *0.4* | *p=0.151* |
|  | **Severe** | *0.8* | *0.4* | *p<0.001* |
|  | **Critical** | *0.5* | *0.2* | *p=0.028* |
| **Chen, et al.^36^**  (N=21) | **Moderate** | *1.1* | *1.0-1.2* | *p=0.049* |
|  | **Severe** | *0.7* | *0.5-0.9* | *p=0.049* |
| **Chen, et al.^41^**  (N=274) | **Survivors** | *1.0* | *0.7-1.4* | *not documented* |
|  | **Non-survivors** | *0.6* | *0.4-0.7* | *not documented* |
| **Wan, et al.^37^**  (N=135) | **Mild** | *1.2* | *0.8-1.6* | *p<0.0001* |
|  | **Severe** | *0.8* | *0.6-1.0* | *p<0.0001* |
| **Zhang, et al.^38^**  (N=138) | **Non-severe** | *0.8* | *0.6-1.2* | *p=0.048* |
|  | **Severe** | *0.7* | *0.5-1.0* | *p=0.048* |
| **Appendix 1b: Neutrophil Count x 10^9^/L** | | | | |
| **Study** | **Outcomes Measured** | **Median value** | **95% CI** | **p-value** |
| **Huang, et al.^25^**  (N=41) | **No ICU care** | 4.4 | 2.0–6.1 | *P=0.00069* |
|  | **ICU care** | 10.6 | 5.0–11.8 | *P=0.00069* |
| **Wu, et al.^30^**  (N=201) | **Without ARDS** | 3.06 | 2.03-5.56 | *P<0.001* |
|  | **With ARDS (alive)** | 5.91 | 3.39-9.70 | *P=0.14* |
|  | **With ARDS (died)** | 7.43 | 5.15-10.60 | *P=0.14* |
| **Wang, et al.^29^**  (N=138) | **Non-ICU** | 2.7 | 1.9-3.9 | *p<0.001* |
|  | **ICU** | 4.6 | 2.6-7.9 | *p<0.001* |
| **Wang, et al.^18^**  (N=296) | **Survivors** | 3.0 | 2.0-4.4 | *p<0.001* |
|  | **Non-survivors** | 6.4 | 3.2-10.0 | *p<0.001* |
| **Wang, et al.^18^**  (N=44) | **Survivors** | 3.4 | 2.0-5.0 | *p<0.001* |
|  | **Non-survivors** | 5.8 | 5.0-8.4 | *p<0.001* |
| **Yao, et al.^42^**  (N=108) | **Non-severe** | 2.53 | 1.89-3.78 | *p=0.002* |
|  | **Severe (alive)** | 3.33 | 1.99-5.07 | *p=0.002* |
|  | **Severe (died)** | 6.55 | 3.39-9.66 | *p=0.002* |
| **Wang, et al.^43^**  (N=65) | **Mild** | 3.8 | 2.4 | *p=0.020* |
|  | **Severe** | 5.7 | 3.7 | *p<0.001* |
|  | **Critical** | 7.7 | 3.9 | *p=0.060* |
| **Chen, et al.^36^**  (N=21) | **Moderate** | 2.7 | 2.1-3.7 | *p=0.002* |
|  | **Severe** | 6.9 | 4.9-9.1 | *p=0.002* |
| **Chen, et al.^41^**  (N=274) | **Survivors** | 3.2 | 2.4-4.5 | *not documented* |
|  | **Non-survivors** | 9.0 | 5.4-12.7 | *not documented* |
| **Wan, et al.^37^**  (N=135) | **Mild** | 3.6 | 3.0-3.9 | *p=0.0015* |
|  | **Severe** | 4.1 | 3.1-5.7 | *p=0.0015* |
