# Supplementary material for: Emergency Department Management of COVID-19: An Evidence-Based Approach
Source: West J Emerg Med. 2020 Sep 25;21(6):32–44. doi: 10.5811/westjem.2020.8.48288 (PMC7673887; doi:10.5811/westjem.2020.8.48288)
Supplement: Supplementary file 2 [file wjem-21-32-s002.docx]

| **Appendix 2: D-Dimer (mg/L, ug/ml)** | | | | |
| --- | --- | --- | --- | --- |
| **Study** | **Outcomes Measured** | **Median value** | **95% CI** | **p-value** |
| **Zhou et al.^24^**  (N=191) | **Survivors** | 0.6 | 0.3–1.0 | *p<0.0001* |
|  | **Non-survivors** | 5.2 | 1.5–21.1 | *p<0.0001* |
| **Huang, et al.^25^** (N=41) | **No ICU care** | 0.5 | 0.3–0.8 | *p<0.0042* |
|  | **ICU care** | 2.4 | 0.6–14.4 | *p<0.0042* |
| **Tang, et al.^26^**  (N=183) | **Survivors** | 0.61 | 0.35-1.29 | *p<0.001* |
|  | **Non-survivors** | 2.12 | 0.77-5.27 | *p<0.001* |
| **Gao, et al.^28^**  (N=43) | **Mild disease** | 0.21 | 0.19-0.27 | *p=0.007* |
|  | **Severe disease** | 0.49 | 0.29-0.91 | *p=0.007* |
| **Wu, et al.^30^**  (N=201) | **Without ARDS** | 0.52 | 0.33-0.93 | *p<0.001* |
|  | **With ARDS (alive)** | 0.49 | 0.31-1.18 | *p<0.001* |
|  | **With ARDS (died)** | 3.95 | 1.15-10.95 | *p=0.001* |
| **Wang, et al.^29^**  (N=138) | **Non-ICU** | 0.16 | 0.10-0.28 | *p<0.001* |
|  | **ICU** | 0.41 | 0.19-1.32 | *p<0.001* |
| **Bi, et al.^33^**  (N=113) | **<0.5** | HR 1 | *-* | *p=0.215* |
|  | **>0.5** | HR 1.765 | 0.7-4.3 | *p=0.215* |
| **Wang, et al.^18^**  (N=296) | **Survivors** | 0.2 | 0.1-0.3 | *p<0.001* |
|  | **Non-survivors** | 0.5 | 0.4-1.4 | *p<0.001* |
| **Wang, et al.^18^**  (N=44) | **Survivors** | 0.6 | 0.3-1.1 | *p=0.025* |
|  | **Non-survivors** | 1.1 | 0.9-1.6 | *p=0.025* |
| **Zou, et al.^34^**  (N=303) | **Mild disease** | 0.43 | 0.31-0.77 | *p<0.001* |
|  | **Severe disease** | 1.04 | 0.73-1.72 | *p<0.001* |
| **Yao, et al.^42^**  (N=108) | **Non-severe** | 1.28 | 0.61-2.69 | *p<0.001* |
|  | **Severe (alive)** | 2.16 | 0.98-2.67 | *p<0.001* |
|  | **Severe (died)** | 15.89 | 2.75-81.59 | *p<0.001* |
| **Wang, et al.^43^**  (N=65) | **Mild** | 1.6 µg/mL | 3 | *p=0.081* |
|  | **Severe** | 4.7 µg/mL | 7.4 | *p<0.001* |
|  | **Critical** | 6.9 µg/mL | 8.4 | *p=0.086* |
| **Liu, et al.^46^**  (N=76) | **Mild** | 0.26 | 0.16-0.45 | *p<0.001* |
|  | **Severe** | 1.0 | 0.33-2.42 | *p<0.001* |
| **Chen, et al.^36^**  (N=21) | **Moderate** | 0.3 | 0.3-0.4 | *p=0.029* |
|  | **Severe** | 2.6 | 0.6-18.7 | *p=0.029* |
| **Chen, et al.^41^**  (N=274) | **Survivors** | 0.6 | 0.3-1.3 | *not documented* |
|  | **Non-survivors** | 4.6 | 1.3-21.0 | *not documented* |
| **Wan, et al.^37^**  (N=135) | **Mild** | 0.3 | 0.2-0.5 | *p<0.0001* |
|  | **Severe** | 0.6 | 0.4-1.1 | *p<0.001* |
| **Han, et al.^27^**  (N=94) | **Ordinary** | 2.14 mg/L | + 2.88 | *p<0.05* |
|  | **Severe** | 19.11 mg/L | + 35.48 | *p<0.01* |
|  | **Critical** | 20.04 mg/L | + 32.39 | *p<0.001* |
| **Zhang, et al.^38^**  (N=138) | **Non-severe** | 0.2 | 0.1-0.3 | *p<0.001* |
|  | **Severe** | 0.4 | 0.2-2.4 | *p<0.001* |
