# Supplementary material for: Emergency Department Management of COVID-19: An Evidence-Based Approach
Source: West J Emerg Med. 2020 Sep 25;21(6):32–44. doi: 10.5811/westjem.2020.8.48288 (PMC7673887; doi:10.5811/westjem.2020.8.48288)
Supplement: Supplementary file 3 [file wjem-21-32-s003.docx]

| **Appendix 3a: Lactate Dehydrogenase (LDH)** | | | | |
| --- | --- | --- | --- | --- |
| **Study** | **Outcomes Measured** | **Median value** | **95% CI** | **p-value** |
| **Zhou et al.^24^**  (N=191) | **Survivors** | 253.5 | 219.0–318.0 | *p<0·0001* |
|  | **Non-survivors** | 521.0 | 363.0–669.0 | *p<0·0001* |
| **Huang, et al.^25^**  N=41) | **No ICU care** | 281.0 | 233.0–357.0 | *P=0.0044* |
|  | **ICU care** | 400.0 | 323.0–578.0 | *P=0.0044* |
| **Wu, et al.^30^**  (N=201) | **Without ARDS** | 257 | 211-320.6 | *P<0.001* |
|  | **With ARDS (alive)** | 349.5 | 293.5-416.0 | *P=0.001* |
|  | **With ARDS (died)** | 484 | 351.0-568.5 | *P=0.001* |
| **Wang, et al.^29^**  (N=138) | **Non-ICU** | 212 | 171-291 | *P<0.001* |
|  | **ICU** | 435 | 302-596 | *p<0.001* |
| **Wang, et al^18^**  (N=296) | **Survivors** | 213.0 | 175.5-256.0 | *p<0.001* |
|  | **Non-survivors** | 478.6 | 363.5-637.2 | *p<0.001* |
| **Wang, et al^18^**  (N=44) | **Survivors** | 327.0 | 207.0-410.0 | *p=0.015* |
|  | **Non-survivors** | 466.5 | 363.5-543.0 | *p=0.015* |
| **Chen, et al.^36^**  (N=21) | **Moderate** | 224.0 | 200.3-251.8 | *p=0.001* |
|  | **Severe** | 537.0 | 433.5-707.5 | *p=0.001* |
| **Chen, et al.^41^**  (N=274) | **Survivors** | 268.0 | 214.3-316.5 | *not documented* |
|  | **Non-survivors** | 564.5 | 431.0-715.8 | *not documented* |
| **Wan, et al.^37^**  (N=135) | **Mild** | 212 | 179.5-259 | *p<0.0001* |
|  | **Severe** | 309 | 253.8-408.3 | *p<0.0001* |
| **Appendix 3b: C-Reactive Protein (mg/L)** | | | | |
| **Study** | **Outcomes Measured** | **Median value** | **95% CI** | **p-value** |
| **Gao, et al.^28^**  (N=43) | **Mild disease** | 18.76 | (+ 22.20) | *p=0.011* |
|  | **Severe disease** | 39.37 | (+ 27.68) | *p=0.011* |
| **Yao, et al.^42^**  (N=108) | **Non-severe** | 6.51 | 0.59-22.7 | *p=0.004* |
|  | **Severe (alive)** | 25.9 | 16.0-34.8 | *p=0.004* |
|  | **Severe (died)** | 39.3 | 30.5-45.2 | *p=0.004* |
| **Wang, et al.^43^**  (N=65) | **Mild** | 53.6 | 57.7 | *p=0.022* |
|  | **Severe** | 91.8 | 77.8 | *p=0.002* |
|  | **Critical** | 114.9 | 62.5 | *p=0.086* |
| **Chen, et al.^36^**  (N=21) | **Moderate** | 22.0 | 14.7-119.4 | *p=0.003* |
|  | **Severe** | 139.4 | 86.9-165.1 | *p=0.003* |
| **Chen, et al.^41^**  (N=274) | **Survivors** | 26.2 | 8.7-55.8 | *not documented* |
|  | **Non-survivors** | 113.0 | 69.1-169.4 | *not documented* |
| **Wan, et al.^37^**  (N=135) | **Mild** | 7.7 | 1.9-31.1 | *p<0.0001* |
|  | **Severe** | 91 | 52.7-136.3 | *p<0.0001* |
| **Zhang, et al.^38^**  (N=138) | **Non-severe** | 28.7 | 9.5-52.1 | *p<0.001* |
|  | **Severe** | 47.6 | 20.6-87.1 | *p<0.001* |
| **Appendix 3c: Ferritin (μg/L)** | | | | |
| **Study** | **Outcomes Measured** | **Median value** | **95% CI** | **p-value** |
| **Zhou et al.^24^**  (N=191) | **Survivors** | 503.2 | 264.0–921.5 | *p<0·0001* |
|  | **Non-survivors** | 1435.3 | 728.9–2000.0 | *p<0·0001* |
| **Wu, et al.^30^**  (N=201) | **Without ARDS** | 457.66 | 223.73-702.65 | *p<0.001* |
|  | **With ARDS (alive)** | 853.00 | 330.33-1968.57 | *p=0.34* |
|  | **With ARDS (died)** | 1096.21 | 559.41->2000 | *p=0.34* |
| **Wang, et al.^43^**  (N=65) | **Mild** | 821.1 | 651.9 | *p=0.094* |
|  | **Severe** | 1331 | 1205 | *p=0.003* |
|  | **Critical** | 1368 | 638.8 | *p=0.342* |
| **Chen, et al.^36^**  (N=21) | **Moderate** | 337.4 | 286.2-1275.4 | *p=0.049* |
|  | **Severe** | 1598.2 | 1424.6-2-36.0 | *p=0.049* |
| **Chen, et al.^41^**  (N=274) | **Survivors** | 481.2 | 265.1-871.5 | *not documented* |
|  | **Non-survivors** | 1418.3 | 915.4-2236.2 | *not documented* |
